# Paralysis of the Hand and Forearm, Caused by Esmarch’s Bloodless Method

**Published:** 1874-07

**Authors:** Robert F. Weir

**Affiliations:** Surgeon to the Roosevelt and St. Luke’s Hospital, New York


### Paralysis of the Hand and Forearm caused by Esmarch's Bloodless Method.

BY ROBERT F. WEIR, M. D.,

Surgeon to the Roosevelt and St. Luke's Hospital, New York.

Since giving in the Record for February 2d, a brief account of the results of Esmarch's bloodless operations, a further experience has not only confirmed its usefulness, but also has brought into view a disadvantage which fortunately, however, can easily be obviated.

The occurrence of paralysis, from the pressure of the rubber cord and tubing upon the nerve trunks, was not alluded to by Esmarch and was brought to my notice in the case of a young man aged 27, whose left elbow joint I had excised by the single incision, at the Roosevelt Hospital, in June, 1873, for chronic arthritis, and in whom motion of the forearm on the arm was admirably perfect; yet as there existed sinuses over the ends of the ulna and humerus, leading to bare bone, the patient was etherized, February 13, 1874, and Esmarch's bandage applied, the rubber cord of a diameter of one quarter.inch being, as usual, tightly drawn three times around the limb at the junction of the upper and middle third of the arm, and the proposed operation performed.

The compression was continued about three-quarters of an hour, and the wounds made by enlarging the sinuses were dressed with charpie and gauze bandage.

After the inflamatory reaction of the wound had passed away, it was found that the patient was unable to flex or extend any of the fingers or the hand on the forearm. He complained of numbness in the tips of all his fingers, of the palm and anterior surface of the forearm. The motor paralysis remained unchanged to March 20, when he was kindly seen by Dr. Seguin, but the anæsthesia had yielded to hyperæsthesia in the dorsum of the fingers, though there was still abolition of tactile sensibility. The examination showed that the lesion was mainly confined to the median nerve. Since that time he has rapidly regained the use of the affected muscles under the use of the galvanic current.

Since recognizing in this way the possibility of such an accident, in the use of Esmarch's method, I have found in the *Medical Times and Gazette*, of January 24, 1874, three cases reported by Langenbeck, where paralysis had occurred in the median nerve after the use of Esmarch's method. Two of the operations performed were for pseudo-arthrosis, and one for necrosis; the resulting paralysis in two cases lasted two weeks, and in the other case it had continued three weeks, when he was discharged (cured presumably). Langenbeck, in consequence of this mishap, recommends that instead of using rubber tubing to compress the artery, an independent elastic bandage be used like those employed in forcing the blood out of the limb, and that it be fastened around the limb, after being drawn tightly, by a pin. In this manner the pressure on the nerve is distributed over a broader surface and an injury of it more successfully avoided. The prolongation of the paralysis, in the case narrated above, is probably due to the narrowness and hardness of the rubber cord employed, as tubing flattens decidedly when applied, and makes thereby a broader pressure.

There is another situation where the same caution should be observed, and that is in the upper part of the leg, where the external peroneal nerve is readily accessible to pressure; though no instance of its paralysis has yet come to my notice.

It is possible, also, that accidents may result in atheromatous patients, from the increased arterial tension produced by the forced addition of the blood belonging to a limb, to the general circulation. Attention to this source of danger has been called by Mahomed, in the *Medical Times and Gazette* of August 9, 1873, in an article on the use of the sphygmograph in aneurism, in which he has shown that the general arterial tension is much augmented when compression is resorted to in the treatment of aneurism.—*Medical Record.*

